# Effects of hypoxia on exercise‐induced diaphragm fatigue in healthy males and females

**DOI:** 10.14814/phy2.15589

**Published:** 2023-01-25

**Authors:** Paige A. Reinhard, Bruno Archiza, Joseph F. Welch, Jenna Benbaruj, Jordan A. Guenette, Michael S. Koehle, A. William Sheel

**Affiliations:** ^1^ School of Kinesiology The University of British Columbia Vancouver British Columbia Canada; ^2^ Department of Physical Therapy Federal University of São Carlos São Carlos SP Brazil; ^3^ Breathing Research and Therapeutics Center, Department of Physical Therapy University of Florida Gainesville Florida USA; ^4^ School of Sport, Exercise and Rehabilitation Sciences University of Birmingham Birmingham UK; ^5^ Department of Physical Therapy The University of British Columbia Vancouver British Columbia Canada; ^6^ Centre for Heart Lung Innovation, Providence Research The University of British Columbia and St. Paul's Hospital Vancouver British Columbia Canada

**Keywords:** cervical magnetic stimulation, inspiratory muscles, sex‐difference

## Abstract

Following high‐intensity, normoxic exercise there is evidence to show that healthy females, on average, exhibit less fatigue of the diaphragm relative to males. In the present study, we combined hypoxia with exercise to test the hypothesis that males and females would develop a similar degree of diaphragm fatigue following cycle exercise at the same relative exercise intensity. Healthy young participants (*n* = 10 male; *n* = 10 female) with a high aerobic capacity (120% predicted) performed two time‐to‐exhaustion (TTE; ~85% maximum) cycle tests on separate days breathing either a normoxic or hypoxic (FiO_2_ = 0.15) gas mixture. Fatigue of the diaphragm was assessed in response to cervical magnetic stimulation prior to, immediately post‐exercise, 10‐, 30‐, and 60‐min post‐exercise. Males and females had similar TTE durations in normoxia (males: 690 ± 181 s; females: 852 ± 401 s) and hypoxia (males: 381 ± 160 s; females: 400 ± 176 s) (*p* > 0.05). Cycling time was significantly shorter in hypoxia versus normoxia in both males and females (*p* < 0.05) and did not differ on the basis of sex (*p* > 0.05). Following the hypoxic TTE tests, males and females experienced a similar degree of diaphragm fatigue compared to normoxia as shown by 20%–25% reductions in transdiaphragmatic twitch pressure. This occurred despite the fact that exercise time in hypoxia was substantially shorter relative to normoxia and the cumulative diaphragm work was lower. We also observed that females did not fully recover from diaphragm fatigue in hypoxia, whereas males did (*p* < 0.05). Sex differences in the rate of diaphragm contractility recovery following exercise in hypoxia might relate to sex‐based differences in substrate utilization or diaphragm blood flow.

## Graphical abstract

Following high‐intensity exercise performed in hypoxia the magnitude of exercise‐induced diaphragm fatigue is similar in males and females. We found that fatigue of the diaphragm persisted significantly longer into recovery versus males. Further study is required to understand the mechanism(s) of sex‐based differences in the rate of diaphragm contractility recovery following exercise in hypoxia.

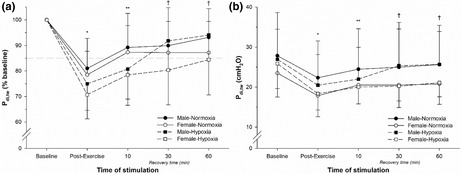

## 1 INTRODUCTION

The mechanical and metabolic costs of sustaining ventilation during dynamic whole‐body exercise are substantial. Exercise places demands on the respiratory musculature such that significant increases in O_2_ transport and blood flow are required to sustain contractions that power adequate changes in alveolar ventilation to match metabolic demand. The diaphragm, as the primary inspiratory muscle, has been shown to fatigue following whole‐body exercise performed at or above 85% maximal O_2_ uptake (V̇O_2max_) (Archiza et al., 2018; Johnson et al., 1993). Factors contributing to the development of diaphragm fatigue in healthy adults relate to a muscle force output versus blood flow/O_2_ transport availability mismatch (Romer & Polkey, 2008; Sheel et al., 2018; Supinski et al., 1988). The reduced O_2_ availability associated with breathing hypoxic air results in reduced maximal exercise work rates, impaired endurance exercise performance, and increased rate of development of both locomotor and respiratory muscle fatigue (Amann et al., 2006; Calbet et al., 2003; Romer et al., 2007). Diaphragm fatigue has been reported following strenuous exercise in both normobaric and hypobaric hypoxia (Babcock et al., 1995; Gudjonsdottir et al., 2001; Vogiatzis et al., 2007). Superimposed hypoxia to heavy submaximal exercise hastens diaphragm fatigue development and delays recovery despite shorter exercise times versus normoxia (Babcock et al., 1995). It has been suggested that the combined effects of hypoxia and heavy exercise may impair diaphragm force generating capacity via: (i) increased ventilation/diaphragmatic work, (ii) lowered O_2_ transport/delivery, and (iii) greater concentration of circulating metabolites (Babcock et al., 1995; Bigland‐Ritchie & Vollestad, 1988; Verges et al., 2010).

There is a growing body of evidence demonstrating that females develop less muscle fatigue than males when performing isometric or dynamic contractions of similar or equal intensity in normoxic conditions [for review see: (Hunter, 2016; Kent‐Braun et al., 2012)]. The reported differences are task specific (i.e., isolated inspiratory work vs. whole‐body exercise); variables that can alter sex differences in fatigability include: the type, intensity and speed of contraction, the muscle group assessed, and environmental conditions (Hunter, 2014). During tasks of isolated inspiratory work performed to equal relative intensities, we (Welch et al., 2018b) and others (Smith et al., 2016) observed attenuated heart rate and arterial blood pressure responses (i.e., blunted respiratory muscle metaboreflex) in females compared to males; however, when matched for absolute work, the magnitude of diaphragm fatigue does not differ between sexes in normoxia (Geary et al., 2019). In contrast and most recently, we observed a higher degree of diaphragm fatigue in females versus males following inspiratory pressure‐threshold loading at equal absolute intensities in acute hypoxia (Archiza et al., 2020). On the other hand, we have previously reported that following heavy cycling exercise, highly trained female cyclists exhibit less diaphragm fatigue versus males in normoxic conditions (Guenette et al., 2010). In the present study, we further explored the effects of hypoxia on diaphragm fatigue in males and females during whole‐body exercise in hypoxia to determine if previous findings are task specific and still present when respiratory and locomotor muscles compete for cardiac output. We hypothesized that males and females would develop a similar degree of diaphragm fatigue during cycle exercise at the same relative exercise intensity in acute hypoxia due to the effects of arterial hypoxemia on muscle force production outweighing any sex‐related difference in muscle fatigability (Amann et al., 2007).

## 2 METHODS

### 2.1 Participants

Twenty healthy participants (10 male, 10 female) volunteered to participate in the study. All participants provided written informed consent, and procedures were approved by The University of British Columbia Research Ethics Board (approval number H18‐02674), which adheres to the *Declaration of Helsinki*, except for registration in a database. All participants were non‐smokers, had pulmonary function within 80% of predicted values (Quanjer et al., 2012), body mass index of 18–30 kg·m^−2^, peak aerobic power ≥ 80% predicted (Jones et al., 1985), and resided at sea level. No participants were excluded for a history of cardiorespiratory disease or any contraindications to exercise testing. Female participants were tested randomly throughout their menstrual cycle (Macnutt et al., 2012; McNulty et al., 2020). All female participants experienced normal menstrual cycles as determined by self‐evaluation via a menstrual cycle questionnaire. Usage of hormonal contraceptives was not an exclusion criterion (Elliott‐Sale et al., 2020).

### 2.2 Experimental protocol

Participants reported to the laboratory on three occasions, separated by >48 h. Prior to all three visits, participants refrained from heavy exercise, caffeine and food consumption for 24, 12, and 2 h, respectively. During the first visit, pulmonary function testing was performed followed by a maximal graded exercise test on a cycle ergometer. Visits two and three were double‐blinded and randomized. Experimental visits two and three were scheduled at the same time of day. Participants performed a time‐to‐exhaustion (TTE) cycle test breathing either a normoxic (FiO_2_ = 0.21, balance N_2_) or hypoxic (FiO_2_ = 0.15, balance N_2_) gas mixture. Cervical magnetic stimulation (CMS) was used to assess diaphragm contractile function prior to, immediately (within 3 min) post‐exercise, 10‐, 30‐, and 60‐min post‐exercise. These time points were selected in order permit comparisons with our previous work (Guenette et al., 2010). Handgrip strength of the dominant hand was measured pre‐ and post‐exercise to determine behavioral changes (e.g., motivation) that may influence maximal voluntary contractions.

### 2.3 Pulmonary function

Spirometry, whole‐body plethysmography, and single‐breath diffusion capacity for carbon monoxide were assessed using a pulmonary function testing system (Vmax Encore 229 with V62J Autobox; CareFusion, Yorba Linda, USA) according to standard procedures (Graham et al., 2019; Macintyre et al., 2005). Pulmonary function measurements were expressed in absolute and percent predicted values (Crapo et al., 1982; Quanjer et al., 2012). Blood hemoglobin concentration ([Hb]) was determined (HemoCue, Helsingborg, Sweden) to correct diffusion capacity measures. Measures of pulmonary function were performed in order to ensure that male and female participants were comparable.

### 2.4 Graded exercise test

Participants completed a 5‐min warm‐up at a self‐selected work rate on an electronically braked cycle ergometer (Velotron, RacerMate). Males and females began cycling at 120 W and 80 W, respectively, at their preferred cadence. Work rate increased in a stepwise fashion by 20 W every 2 min. The test was terminated when cadence dropped below 60 rpm despite verbal encouragement. During exercise, participants breathed through a customized two‐way non‐rebreathing valve (2700B, Hans‐Rudolph) attached to a mixing chamber. Mixed expired gases were sampled using a calibrated O_2_/CO_2_ gas analyzer (ML206 Gas Analyzer, ADInstruments). Inspired and expired airflows were measured using calibrated pneumotachographs (no. 3813, Hans Rudolph) and volume was determined by numerical integration of inspiratory and expiratory flow. Heart rate was measured with a commercially available chest monitor (T34, Polar, Electro).

### 2.5 Cycling time to exhaustion

Constant‐load TTE cycling tests in normoxic and acute hypoxic conditions were conducted at a relative work rate corresponding to 60% of the difference between the calculated gas exchange threshold and peak work rate from the graded cycling test (Archiza et al., 2018; Lansley et al., 2011). A standardized 2 min warm‐up (1 min at 40% and 1 min at 60% V̇O_2max_) was performed prior to each TTE test. Participants inspired specific gas mixtures for normoxic and hypoxic (F_I_O_2_ = 0.21 and 0.15, respectively) conditions during cycle exercise only. Cardiorespiratory variables were collected using the same hardware and software as the graded exercise test. Prior to commencing exercise, participants were instrumented with two balloon‐tipped catheters to determine esophageal and gastric pressures (P_eso_ and P_gas_, respectively) (details below). A finger pulse oximeter (9600 Pulse Oximeter, Nonin, Minnesota, USA) was used to assess peripheral oxygen saturation (S_p_O_2_). Blood lactate concentration was obtained from a fingertip every 2 min and at end exercise (The Edge, Woodley Equipment). Testing was terminated when cadence was <60 rpm. No verbal encouragement was provided to participants during the TTE exercise tests to ensure consistency between experimental trials.

### 2.6 Pressure and electromyography

Pressure and electromyographic measurements have been described in detail previously (Archiza et al., 2020). Briefly, P_eso_ and P_ga_ were assessed by inserting two balloon‐tipped catheters (no. 47‐9005, Ackrad Laboratory) intranasally into the esophagus and stomach, respectively. Both balloons were initially inserted into the stomach and air was evacuated by a Valsalva maneuver. Esophageal and gastric balloons were filled with 1 and 2 ml of air, respectively. The esophageal balloon was then displaced into the lower one‐third of the esophagus and placement verified using the occlusion method (Baydur et al., 1982). Transdiaphragmatic pressure (P_di_) was calculated as the difference between P_gas_ and P_eso_. Mouth pressure (P_mo_) was measured through a port in the mouthpiece. Pressures were measured continuously using calibrated (2021P, Digitron) differential pressure transducers (model DP15‐34, 31 Validyne Engineering). Surface electromyography (EMG) of the right and left hemidiaphragm were recorded using self‐adhesive Ag/AgCl electrodes (H59P, Kendall‐LTP) (Merletti et al., 2001). EMG signals were amplified, band‐pass filtered (0.1 Hz to 3 kHz; P511 Series, Grass Instruments), and digitized for subsequent analysis (LabChart v8.1.17, ADInstruments).

### 2.7 Diaphragm fatigue

Cervical magnetic stimulation was used to assess non‐volitional diaphragm contractility as described elsewhere (Similowski et al., 1989; Welch et al., 2017, 2018a). Briefly, using C_7_ as a landmark, a 90‐mm circular coil (P/N 9784–00, Magstim, Whitland, Wales) powered by a magnetic stimulator (Magstim 200 Mono Pulse, MagStim) was used to stimulate the phrenic nerve roots. The coil was moved slightly from C_7_ to optimize signal of evoked potentials. All stimuli were performed at functional residual capacity, which was verified by monitoring P_eso_ and P_gas_ immediately preceding stimulations. To determine if the phrenic nerves were maximally activated, three stimuli at increasing stimulator output intensity (60, 70, 80, 90, 95, and 100%) were delivered with each stimulation separated by 30 s. Diaphragm fatigue was quantified using a series of potentiated (delivery of stimulus preceded by ~5 s maximal inspiratory effort) twitches delivered at 100% of the maximal stimulator output at baseline and following exercise. Participants were given visual feedback on the maximal inspiratory efforts and coaching from experimenters on diaphragm recruitment. For the purposes of our study, fatigue of the diaphragm was assumed present if there was a ≥15% reduction in transdiaphragmatic twitch pressure (P_di,tw_) relative to resting baseline levels (Guenette et al., 2010; Welch et al., 2018b). This definition of fatigue is based on an approximate two‐ to threefold increase in the coefficient of variation of P_di,tw_.

### 2.8 Data analysis

Five blocks of stimuli were delivered (pre‐, immediately post‐exercise [within 3 min], 10‐, 30‐, and 60‐min into recovery) with each block containing 7–8 twitches; the first 3–4 were discarded, and the latter 4 were used for analysis. Participants breathed room air (F_I_O_2_ = 0.21) during recovery from both the hypoxia and normoxia trials. Pressure–time products (PTP) were calculated by integrating the area of pressure curves (P_eso_, P_gas_, and P_di_) during inspiration for every minute of exercise. Slope calculation (%TTE in 20% increments) of PTP_di_:PTP_eso_ was used to estimate diaphragm contribution to overall respiratory muscle force production. Baseline maximal inspiratory pressure or inspiratory capacity maneuvers were used to normalize EMG of the sternocleidomastoid (EMG_SCM_) during TTE. Diaphragm compound muscle action potentials were analyzed for: peak‐to‐peak twitch amplitude, onset latency, duration, and total rectified area using custom software (MATLAB R2015a, MathWorks).

### 2.9 Statistical analysis

A three‐way (sex [male vs. female], condition [normoxia vs. hypoxia], and time [20, 40, 60, 80, and 100% TTE]) repeated measures analysis of variance (ANOVA) was used to make comparisons for cardiorespiratory variables during exercise. Variables associated with diaphragm fatigue were assessed using a three‐way (sex [male vs. female], condition [normoxia vs. hypoxia], and time [within 3‐, 10‐, 30‐, and 60‐min post‐exercise]) repeated measures ANOVA. In the case of a significant main effects or interactions, differences were further investigated with Tukey post‐hoc tests. Sex differences in participant characteristics, maximal exercise data, and diaphragm compound muscle action potential characteristics were tested by independent student's t‐tests. Repeated measures ANOVA was used to determine if the phrenic nerves were maximally activated by comparing P_di,tw_ at all submaximal intensities of stimulation (60, 70, 80, 90, 95%) with the maximal stimulator output (100%). On an individual basis, a plateau was considered present if the average P_di,tw_ at submaximal and maximal stimulation intensities was separated by equal to or less than the within‐block coefficient of variation for all twitches. Statistical significance was set at *p* ≤ 0.05 for all comparisons. Statistical analyses were performed using JASP (v0.12.1) and Jamovi (v.2.3.12.). Values are presented at mean ± SD.

## 3 RESULTS

### 3.1 Participant characteristics

Pulmonary function and maximal exercise values are presented in Table 1. Males and females were of similar height, mass, age, pulmonary function (based on predicted values) and V̇O_2max_.

**TABLE 1:** Participant characteristics, pulmonary function and maximal exercise data

	Female (*n* = 10)	Male (*n* = 10)
Age, years	24 ± 2	25 ± 3
Height, cm	175 ± 7	179 ± 5
Body mass, kg	69.5 ± 9.2	75.0 ± 5.9
Pulmonary Function		
FVC, liters	4.85 ± 0.94	5.90 ± 0.75*
FVC, % predicted	107.8 ± 17.8	106.7 ± 11.3
FEV_1_, liters	3.89 ± 0.94	4.75 ± 0.54*
FEV_1_, % predicted	100.6 ± 13.7	101.3 ± 9.5
FEV_1_/FVC, %	82.1 ± 6.1	81.7 ± 3.2
FEV_1_/FVC, % predicted	94.1 ± 7.9	96.3 ± 6.7
FEF_25‐75_, liters·s^−1^	4.0 ± 0.6	4.7 ± 0.9*
FEF_25‐75_, % predicted	94.8 ± 17.1	92.9 ± 12.2
PEF, liters·s^−1^	8.3 ± 1.2	12.4 ± 2.5*
PEF, % predicted	107.6 ± 16.1	120.1 ± 24.8
DLCO, ml·mg^−1^·mmHg^−1^	29.5 ± 4.4	40.5 ± 4.5*
DLCO, % predicted	98.0 ± 13.6	103.5 ± 11.1
Hb, g·dl^−1^	13.2 ± 0.6	14.2 ± 1.1*
TLC, liters	6.4 ± 0.9	7.4 ± 0.6*
TLC, % predicted	92.1 ± 11.0	96.5 ± 7.3
Maximal Exercise Data		
HR, beats·min^−1^	187 ± 12	181 ± 5
V̇O_2_, ml·min^−1^·kg^−1^	45.4 ± 10.6	54.0 ± 9.9*
V̇O_2_, liters·min^−1^	3.14 ± 0.70	4.03 ± 0.70*
V̇O_2_, % predicted	119.7 ± 28.1	118.7 ± 17.5
V̇CO_2_, liters·min^−1^	3.30 ± 0.7	4.31 ± 0.84*
RER	1.08 ± 0.05	1.07 ± 0.06
V̇_E_, liters·min^−1^	113.0 ± 28.1	159.3 ± 39.1*
Fb, breaths·min^−1^	56.0 ± 8.4	54.4 ± 10.9
Vt, liters	2.06 ± 0.43	2.76 ± 0.36*
Maximal Workload, W	244 ± 51	306 ± 60*

Abbreviations: FVC, forced vital capacity; FEV_1_, forced expiratory volume in 1‐s; FEF_25‐75_, forced expiratory flow between 25%–75% of FVC; PEF, peak expiratory flow; DLCO, diffusion capacity of the lungs for carbon monoxide; TLC, total lung capacity; Hb, hemoglobin concentration; HR, heart rate; V̇O_2_, oxygen uptake; V̇CO_2_, carbon dioxide output; RER, respiratory exchange ratio; V̇_E_, minute ventilation; F*b*, breathing frequency; Vt, tidal volume. Values are mean ± SD. *Significantly different from females (*p* < 0.05).

### 3.2 Diaphragm fatigue

**FIGURE 1:**
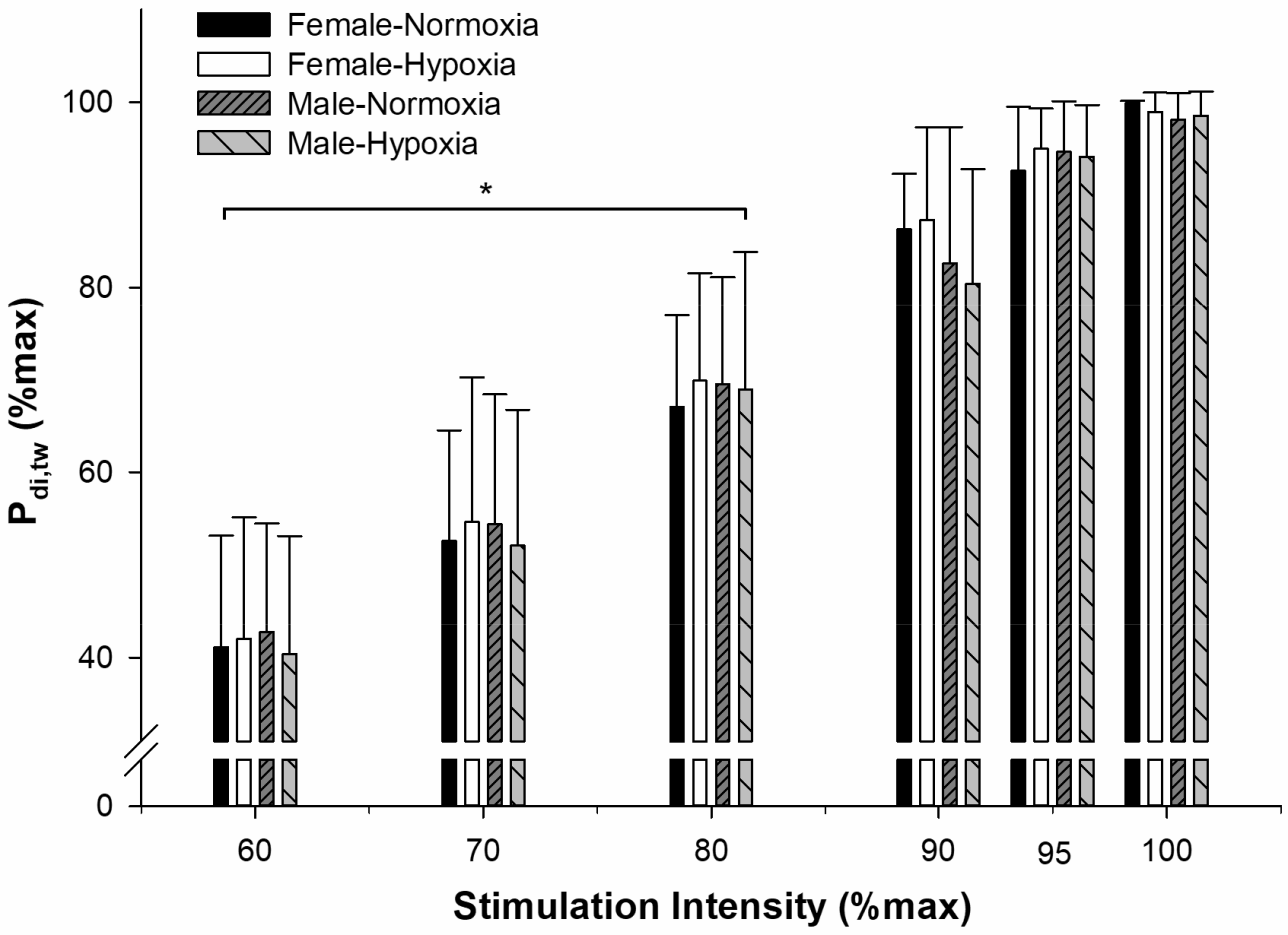
Diaphragm recruitment protocol from 60 to 100% of magnetic stimulator output. Procedure performed for each sex and condition. Values are mean ± SD. *Significantly different from 100% maximal stimulator output in respective condition.

**FIGURE 2:**
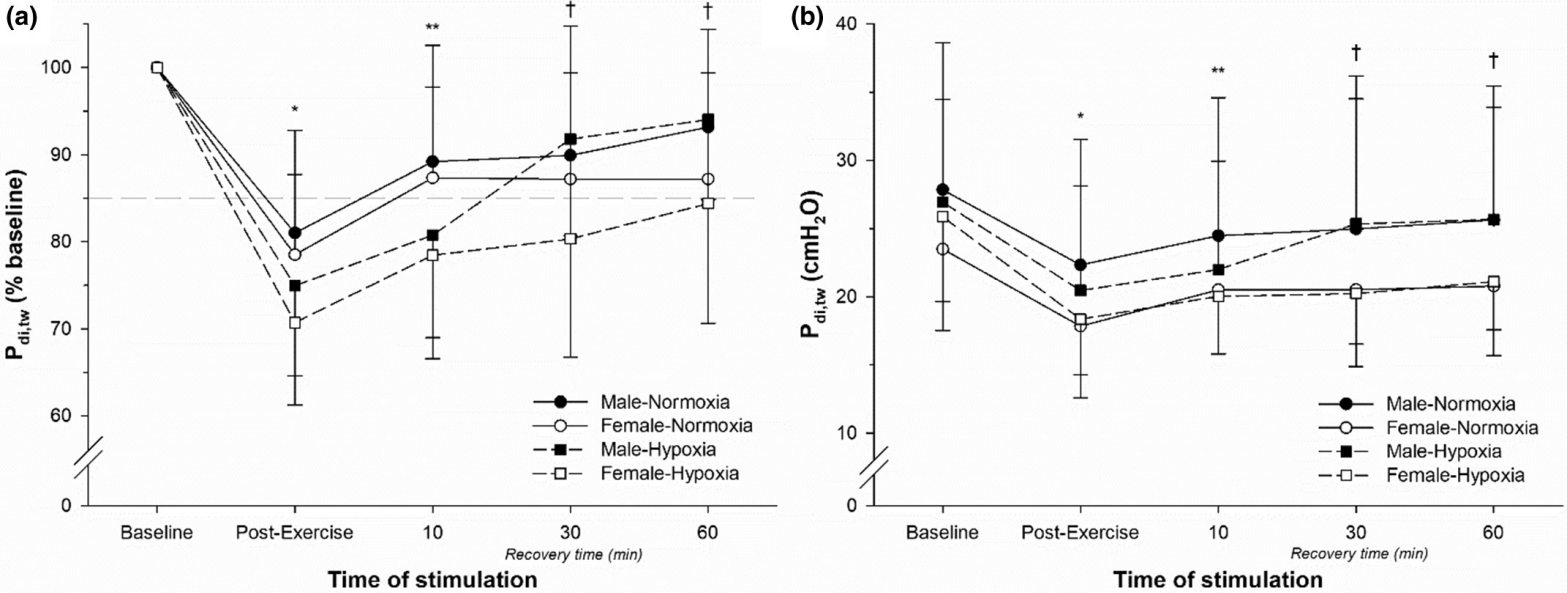
Diaphragm fatigue result from CMS for each sex and condition as (a) relative (percent of baseline) and (b) absolute P_di,tw_ values. Values are mean ± SD. The dashed horizontal line in panel A represents the criterion used to consider presence of diaphragmatic fatigue (15% reduction in P_di,tw_). *Significantly different from baseline for all groups. **Significantly different from baseline for hypoxic groups only. ^†^Significantly different from baseline for females in hypoxia only.

In response to increasing stimulation intensity, P_di,tw_ on average plateaued at 90% of the maximal stimulator output for both males and females (Figure 1). The P_di,tw_ coefficient of variation at baseline was 6.1 ± 2.3% and 5.8 ± 2.5% for males and females, respectively. The relative (% of baseline) and absolute (cmH_2_O) changes in P_di,tw_ pre‐ vs. post‐exercise in normoxia and hypoxia are illustrated in Figure 2a,b, respectively. To report relative changes (% of baseline), baseline P_di,tw_ values were set to 100% and all recovery values were then individually normalized for each subject. This method was previously shown to represent a good measure of diaphragm fatigability as it considers interindividual differences in diaphragm contractility/response to diaphragm magnetic stimuli (Archiza et al., 2018, 2020; Geary et al., 2019; Guenette et al., 2010; Welch et al., 2017, 2018a, 2018b). Compound muscle action potential characteristics are shown in Table 2 with no significant changes based on sex or inspired gas condition.

**TABLE 2:** Compound muscle action potential characteristics

	Normoxia pre‐test	Normoxia post‐test	Hypoxia pre‐test	Hypoxia post‐test
Male				
Latency (ms)	4.6 ± 0.5	4.6 ± 0.6	4.5 ± 0.4	4.6 ± 0.3
Amplitude (V)	4.2 ± 1.1	4.3 ± 1.0	4.5 ± 0.7	4.6 ± 0.9
Duration (ms)	39.6 ± 6.6	38.4 ± 4.4	38.9 ± 6.0	39.6 ± 4.6
Area (V·ms)	32.8 ± 10.2	33.1 ± 6.2	35.0 ± 6.2	35.4 ± 6.4
Female				
Latency (ms)	4.3 ± 0.4	4.2 ± 0.4	4.5 ± 0.6	4.7 ± 0.6
Amplitude (V)	4.5 ± 0.8	4.3 ± 0.7	4.6 ± 1.2	4.5 ± 1.1
Duration (ms)	37.7 ± 5.2	36.3 ± 7.7	39.7 ± 10.3	39.3 ± 9.1
Area (V·ms)	36.2 ± 5.4	34.4 ± 2.7	32.5 ± 10.3	33.4 ± 9.3

*Note*: No significant differences were observed. Values are mean ± SD.

Following exercise in normoxia, the average reduction in P_di,tw_ for females was 21.5 ± 9.2, 12.7 ± 10.4, 12.8 ± 12.2, and 12.9 ± 9.7% immediately post‐exercise and 10, 30 and 60 min into recovery, respectively. At the same time points, the average reduction in P_di,tw_ for males was 19.0 ± 11.7, 10.8 ± 13.3, 10.1 ± 14.8, and 6.8 ± 11.2%, respectively. Following exercise in hypoxia, the average reduction in P_di,tw_ for females was 29.3 ± 6.1, 21.5 ± 9.5, 19.7 ± 13.6, and 15.6 ± 13.7% at 5, 10, 30, and 60 min post‐exercise, respectively. At the same time points, the average reduction in P_di,tw_ for males was 25.0 ± 9.1, 19.2 ± 14.1, 7.1 ± 12.5, and 5.8 ± 9.1% from baseline.

A main effect of time in change in P_di,tw_ was observed for both males and females in both normoxia and hypoxia (*p* < 0.001). Compared to immediately post‐exercise, P_di,tw_ was increased at 10, 30, and 60 min post‐exercise in both normoxia and hypoxia (*p* = 0.003, *p* = 0.013, *p* < 0.001, respectively). A time by condition interaction effect was also observed (*p* = 0.036). In the hypoxic condition, P_di,tw_ was also significantly lower at 10 min post‐exercise compared to 30 and 60 min post‐exercise in the hypoxic condition (*p* = 0.024, *p* = 0.004), but not the normoxic condition (*p* > 0.05). No main effect of condition or sex was observed (*p* = 0.079, *p* = 0.329, respectively).

### 3.3 Handgrip strength

Voluntary handgrip strength (males normoxia pre: 367 ± 54 N, post: 370 ± 67 N; males hypoxia pre: 359 ± 66 N, post: 355 ± 65 N; females normoxia pre: 265 ± 59 N, post: 239 ± 42 N; females hypoxia pre: 250 ± 55 N, post: 254 ± 48 N) was unchanged from baseline post‐exercise in normoxia and hypoxia in both males and females (all *p* > 0.05).

### 3.4 Time to exhaustion (TTE)

Mean workload during TTE exercise in normoxia and hypoxia was 265 ± 57 W for males and 197 ± 41 W for females. Males and females had no significant differences in TTE durations in normoxia (males: 690 ± 181 s; females: 852 ± 401 s, *p* = 0.501) and hypoxia (males: 381 ± 160 s; females: 400 ± 176 s, *p* = 0.996). Cycling time was significantly shorter in hypoxia versus normoxia in both males and females (*p* = 0.004 and *p* < 0.001, respectively) and did not differ on the basis of sex (*p* = 0.353). Respiratory and cardiovascular responses during TTE exercise are shown in Table 3. A main effect of condition (hypoxia vs. normoxia) was found for minute ventilation (*p* = 0.019), heart rate (*p* = 0.012), V̇O_2_ (*p* = 0.003), and respiratory exchange ratio (*p* < 0.001) during TTE exercise, where all variables showed lower absolute values during hypoxia compared to normoxia; however, minute ventilation, respiratory exchange ratio, heart rate, and V̇O_2_ were not statistically different between conditions at end exercise (all *p* > 0.05).

**TABLE 3:** Cardiorespiratory variables during same relative intensity TTE exercise during normoxic and hypoxic exercise

% TTE	Normoxia (F_I_O_2_ = 0.21)					Hypoxia (F_I_O_2_ = 0.15)				
	20%	40%	60%	80%	100%	20%	40%	60%	80%	100%
Male										
HR, beats·min^−1^	154 ± 10	162 ± 9^a^	169 ± 8^a^	174 ± 7^a^	176 ± 9^a^	147 ± 9	155 ± 8^a^	161 ± 5^a^	168 ± 5 ^a^	167 ± 12^a^
V_T_, liters	2.7 ± 0.6	2.8 ± 0.4	2.7 ± 0.3	2.7 ± 0.4	2.6 ± 0.4	2.8 ± 0.4	2.8 ± 0.4	2.8 ± 0.4	2.8 ± 0.4	2.6 ± 0.3
F*b*, breaths·min^−1^	34 ± 6	37 ± 6	42 ± 7	46 ± 7^a^	49 ± 9^a^	33 ± 5	38 ± 6	42 ± 8^a^	48 ± 9^a^	55 ± 10^a^
V̇_E_, liters·min^−1^	94 ± 36	111 ± 34^a^	118 ± 32^a^	131 ± 33^a^	135 ± 33^a^	99 ± 27	118 ± 36^a^	133 ± 40^a^	147 ± 42^a^	156 ± 40^a^
V̇O_2_, liters·min^−1^	3.36 ± 0.97	3.66 ± 0.99	3.68 ± 0.74	3.79 ± 0.76^a^	3.76 ± 0.79^a^	2.89 ± 0.55	3.15 ± 0.52	3.29 ± 0.46^a^	3.41 ± 0.43^a^	3.52 ± 0.57^a^
%V̇O_2max_, %	83 ± 19	90 ± 17	91 ± 14	94 ± 15^a^	94 ± 16^a^	72 ± 9	79 ± 9	82 ± 11^a^	85 ± 12^a^	88 ± 13^a^
RER	1.01 ± 0.09	1.01 ± 0.08	0.99 ± 0.07	0.99 ± 0.08	0.99 ± 0.08	1.11 ± 0.11	1.17 ± 0.10^b^	1.15 ± 0.11^b^	1.07 ± 0.11	1.06 ± 0.10
SpO_2_, %	98 ± 2	97 ± 2	97 ± 1	95 ± 4	95 ± 4	89 ± 4^b^	87 ± 3^b^	87 ± 3^b^	87 ± 3^b^	84 ± 4^b^
Female										
HR, beats·min^−1^	158 ± 12	164 ± 11	171 ± 11^a^	173 ± 10^a^	173 ± 9^a^	156 ± 11	163 ± 10	164 ± 14^a^	169 ± 12^a^	172 ± 12^a^
V_T_, liters	2.07 ± 0.50	1.95 ± 0.44^c^	1.97 ± 0.36^c^	1.83 ± 0.45^c^	1.77 ± 0.42^a^, ^c^	2.14 ± 0.50	2.07 ± 0.47^c^	1.97 ± 0.45^c^	1.96 ± 0.46^c^	1.97 ± 0.39
F*b*, breaths·min^−1^	38 ± 8	45 ± 8	47 ± 9^a^	53 ± 10^a^	57 ± 1 2^a^	38 ± 6	46 ± 9	50 ± 8^a^	52 ± 8^a^	53 ± 8^a^
V̇_E_, liters·min^−1^	81 ± 26	90 ± 24	96 ± 27^a^	100 ± 31^a^	106 ± 35^a^	85 ± 25	95 ± 24	103 ± 27^a^	107 ± 28^a^	109 ± 28^a^
V̇O_2_, liters·min^−1^	2.75 ± 0.78	2.78 ± 0.77	2.85 ± 0.77	2.91 ± 0.92	2.98 ± 1.04	2.37 ± 0.69	2.43 ± 0.64	2.47 ± 0.61	2.56 ± 0.66	2.56 ± 0.68
%V̇O_2max_, %	87 ± 9	89 ± 10	91 ± 8	92 ± 11	94 ± 16	75 ± 12	77 ± 9	80 ± 11	82 ± 13	82 ± 12
RER	1.00 ± 0.06	1.00 ± 0.06	0.98 ± 0.06	0.97 ± 0.06	0.96 ± 0.06	1.09 ± 0.11	1.11 ± 0.10	1.10 ± 0.08	1.07 ± 0.08	1.07 ± 0.07
SpO_2_, %	97 ± 3	95 ± 4	96 ± 4	96 ± 3	97 ± 2	88 ± 4^b^	87 ± 4^b^	84 ± 4^b^	83 ± 6^b^	83 ± 5^b^

Abbreviations: F_I_O_2_, inspired fraction of oxygen; HR, heart rate; Vt, tidal volume; F*b*, breathing frequency; V̇_E_, minute ventilation; V̇O_2_, oxygen uptake; RER, respiratory exchange ratio; SpO_2_, pulse oximeter oxyhemoglobin saturation. Values are means ± SD.

^a^ Significantly different from the beginning of exercise (20% TTE) within condition.

^b^ Significantly different from the respective values in normoxia.

^c^ Significantly different between males and females in the respective condition and time.

Regardless of exercise condition, males demonstrated larger tidal volumes (*p* < 0.001), V̇O_2_ (*p* = 0.017), and a lower breathing frequency (*p* = 0.046) compared to females. Exercise in hypoxia significantly increased blood lactate concentration compared to normoxia by 4 min into exercise (males normoxia = 6.5 ± 1.8; males hypoxia = 8.7 ± 3.5; females normoxia = 6.6 ± 1.6; females hypoxia = 8.7 ± 1.5 mmol/dl, *p* = 0.037; *p* = 0.043, respectively), but end‐exercise values were not significantly different between sexes or conditions (males normoxia = 10.3 ± 3.2; males hypoxia = 11.6 ± 3.8; females normoxia = 9.8 ± 2.6; females hypoxia = 11.6 ± 2.6 mmol/dl, *p* = 0.694; *p* = 0.119, respectively).

### 3.5 Pressure–time product

**FIGURE 3:**
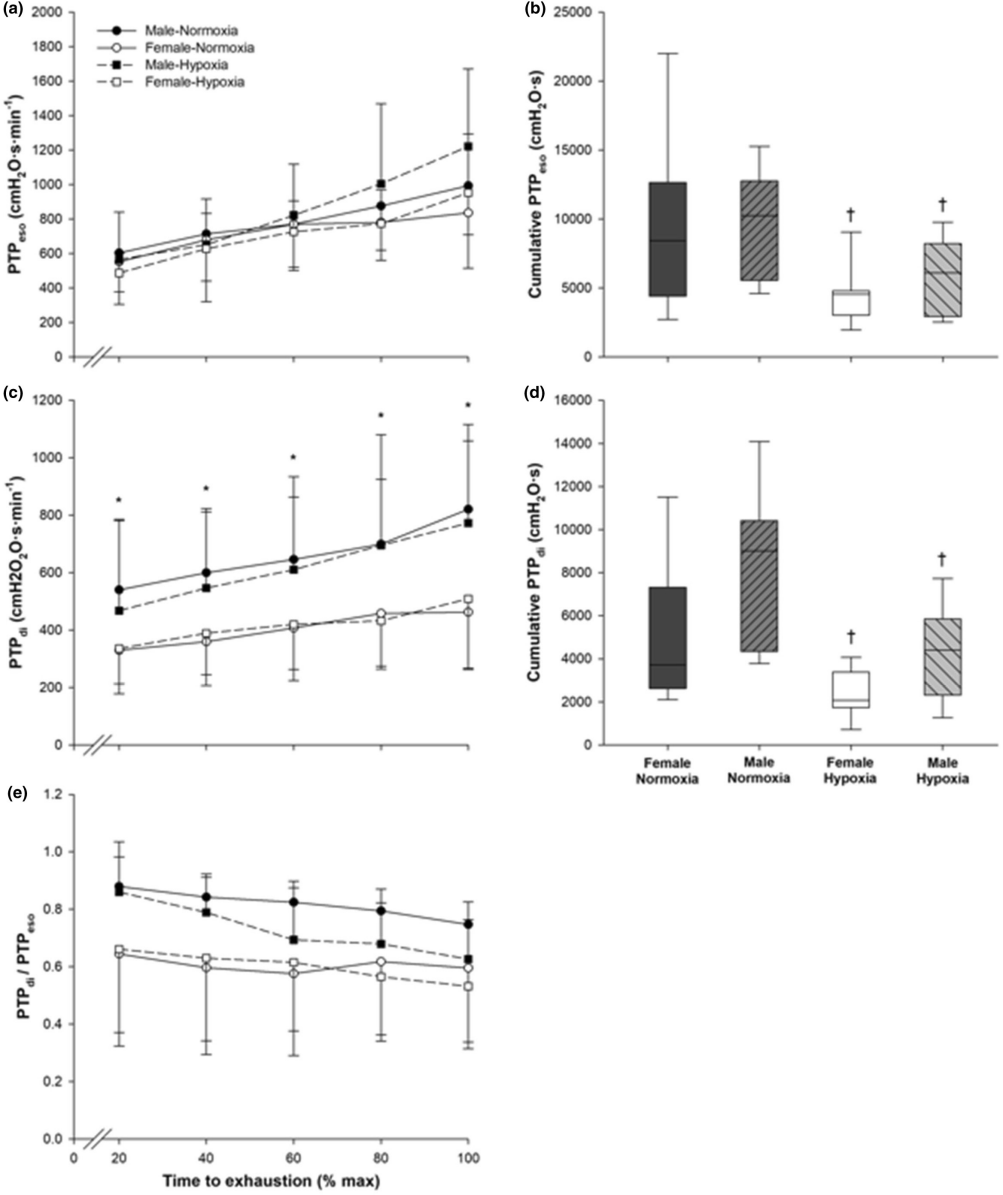
Calculated PTP values from P_eso_, P_ga_, and P_di_. (a) PTP_eso_, (b) cumulative PTP_eso_ at end exercise, (c) PTP_di_, (d) cumulative PTP_di_ at end exercise, and (e) PTP_di_/PTP_eso_. Values are mean ± SD. Box plots (b and d) extend to the 25 and 75 percentiles, whereas the error bars extend to the extreme individual data point in each direction and the horizontal bar indicates the median value. No outliers were identified. *Significant difference between males and females. ^†^Significant difference between normoxia and hypoxia.

PTP_eso_, PTP_di_, and the ratio of PTP_di_:PTP_eso_ are shown in Figure 3a,c,e, respectively, whereas the cumulative PTP_eso_ and PTP_di_ are reported in Figure 3b,d, respectively. A significant main effect of time was observed for PTP_eso_, PTP_gas_, and PTP_di_. PTP_eso_ and PTP_di_ both increased as exercise progressed regardless of sex or FiO_2_ condition (*p* < 0.001), as shown in Figure 3a,c. However, PTP_di_ was significantly greater in males than females regardless of FiO_2_ condition (*p* = 0.019). No significant sex effect was observed in either condition for PTP_eso_ and PTP_ga_.

A significant main effect of time was also shown for PTP_di_:PTP_eso_ (*p* < 0.001). In both conditions, PTP_di_:PTP_eso_ significantly decreased from 20% TTE to 60% (*p* = 0.030), 80% (*p* = 0.022), and 100% TTE (*p* = 0.001). PTP_di_:PTP_eso_ also significantly decreased from 40% TTE to 80% (*p* = 0.038) and 100% TTE (*p* = 0.004). A condition by time interaction effect was also observed (*p* = 0.011); PTP_di_:PTP_eso_ was significantly greater in normoxia than hypoxia at 80% TTE (*p* = 0.006) and 100% TTE (*p* = 0.013). This trend corresponds to the negative PTP_di_:PTP_eso_ slope observed in both the normoxic (males: −0.22, females: −0.04) and hypoxic (males: −0.28, females: −0.17) conditions, as shown in Figure 3e. No significant effect of sex (*p* = 0.051) or condition (*p* = 0.135) was observed for PTP_di_:PTP_eso_ slope.

Cumulative PTP_eso_, cumulative PTP_gas_, and cumulative PTP_di_ were all significantly increased in the normoxic condition than the hypoxic condition (all *p* < 0.001). No significant sex effect was observed in cumulative PTP.

## 4 DISCUSSION

### 4.1 Main findings

We tested the hypothesis that males and females would develop a similar degree of diaphragm fatigue following high‐intensity cycle exercise in hypoxia compared to normoxia. The main findings are two‐fold. First, diaphragm fatigue occurred following exercise to a similar extent in both normoxia and hypoxia. This occurred despite the fact that exercise time in hypoxia was substantially shorter relative to normoxia and the cumulative diaphragm work was lower. Moreover, the degree of diaphragm fatigue at exercise exhaustion did not differ on the basis of sex or F_I_O_2_. Second, the degree of female diaphragm fatigue in hypoxia was similar to that in normoxia, but P_di,tw_ was impaired for significantly longer into recovery versus males. Females exhibited significantly lower P_di,tw_ values from baseline through 60 min into recovery, with males exhibiting significantly lower P_di,tw_ values through 10 min into recovery. Collectively, the findings suggest that diaphragm force production at exercise exhaustion is similar under normoxic and hypoxic conditions for both healthy males and females of moderate levels of aerobic fitness. However, the female diaphragm did not fully recover after hypoxic exercise while the male diaphragm did.

### 4.2 Exercise time to exhaustion and degree of diaphragm fatigue

Performing high‐intensity exercise while breathing a hypoxic inspirate decreases exercise tolerance in healthy humans (Amann et al., 2006; Babcock et al., 1995). Without exception, each participant in the current study had a lower TTE with acute hypoxia versus normoxia. Moderate hypoxia has been shown to decrease maximal cardiac output (Peltonen et al., 2001), whole‐body V̇O_2max_, and increase V̇_E_ (Bigland‐Ritchie & Vollestad, 1988). Both groups in the present study had significantly lower V̇O_2_ values in the hypoxic condition while V̇_E_ was maintained or slightly increased, indicating the pulmonary response was likely maximal. Interestingly, it has been shown that despite significant reductions in the work capacity of limb locomotor muscles following exercise in hypoxia, the work capacity of the respiratory muscles is largely unimpaired (Bigland‐Ritchie & Vollestad, 1988). This was interpreted to mean that the demands of the locomotor muscles during hypoxic exercise must not exceed the capacity of the respiratory system and therefore, the force producing capabilities of exercising muscle are a means of ‘protection’ for the respiratory system.

We found that hypoxia decreased exercise TTE and yet, the degree of diaphragm fatigue was similar to normoxia in both males and females, despite males exercising for >2 min longer than females on average. With respect normoxic exercise, the present findings differ from those of Guenette et al. (2010), who found that highly competitive female cyclists with a high level of aerobic fitness exhibit a resistance to diaphragm fatigue following normoxic cycling to exhaustion. The female participants in the present study were not as well‐trained as those in our previous work (V̇O_2max_ ~ 57 vs. ~45 ml·min^−1^·kg^−1^) (Guenette et al., 2010) and we considered the possibility that differences in training status may have contributed to the observed discrepancy. For example, with training, increased capacity for oxidative phosphorylation may attenuate fatigue development and this could, in turn, contribute to differences between sexes and therefore, between studies. However, others have compared subjects (20 males, 4 females) with high (V̇O_2max_ ~ 70 ml·kg^−1^·min^−1^) and moderate (V̇O_2max_ ~ 50 ml·kg^−1^·min^−1^) levels of aerobic fitness and found no difference in low frequency diaphragm fatigue after whole‐body exercise to exhaustion (Babcock et al., 1996). In addition, the higher relative work rate used by Guenette et al. (2010) and the current study may have contributed to the discrepancy in diaphragm fatigue results. As the intensity of exercise increases, sex differences can become more pronounced due to the increased recruitment of type II muscle fibers, of which males typically have a higher proportion compared to females (Hunter, 2016). It remains unknown what influence aerobic fitness has on exercise‐induced diaphragm fatigue in females.

At the end of the hypoxic TTE tests, both males and females experienced a similar degree of diaphragm fatigue compared to normoxia. Our observations support the previous work of Babcock et al. (2020) who found a similar amount of diaphragm fatigue (in a sample of mixed males and females) at exhaustion in moderate acute hypoxia and normoxia. Participants in both studies cycled significantly less time during the hypoxic trial, as compared to normoxia. The hypoxia‐induced reduction in S_p_O_2_ lowers O_2_ delivery to the muscle and presumably increases the rate of fatigue development (Babcock et al., 1995). Using varying levels of F_I_O_2_ (0.13, 0.17, 0.21 and 0.26, respectively) Vogiatzis et al. (2007) found that a greater amount of diaphragmatic fatigue in hypoxia at lower leg work rates. In the present study, moderate hypoxia (F_I_O_2_ ~ 0.15) appears to not increase the degree of diaphragm fatigue at exhaustion, rather the development of fatigue is accelerated. Moreover, exercising in hypoxia may also lead to an increased production and accumulation of metabolites leading to an increase in sympathetic activation. In turn, this leads to an increased sympathetic vasomotor outflow with subsequent reductions in limb blood flow and exercise capacities (Babcock et al., 1995; Bigland‐Ritchie & Vollestad, 1988; Romer et al., 2007). We have previously made the case for a significant influence of limb locomotor muscle activity on respiratory muscle blood flow and fatigue (Sheel et al., 2018). Here we suggest that the similar degree of diaphragm fatigue following whole‐body exercise in normoxia and hypoxia, despite a lower cumulative work performed by the diaphragm in hypoxia, relates to: (i) increasing concentrations of circulating metabolites that originate from the exercising limb, and (ii) compromised blood flow to the diaphragm. We interpret our observations to mean that exercise in hypoxia further reveals an important influence of limb activity on respiratory muscle blood flow and the development of fatigue.

### 4.3 Recovery from diaphragm fatigue

We observed a prolonged diaphragm fatigue recovery time in the hypoxic condition despite a reduced exercise time, which is consistent with other findings (Amann et al., 2006; Babcock et al., 1995). The present study adds to these observations where we found blunted recovery of P_di,tw_ over time in females versus males. Males experienced a decrease in P_di,tw_ post‐exercise that extended to 10 min post‐exercise. Females experienced a significantly reduced P_di,tw_ up until the end of the measurement period (60 min post‐exercise). Breathing a low F_I_O_2_ during heavy exercise appears to not change the degree of diaphragm fatigue at exhaustion but prolongs muscle contractility recovery. Previous research (Fregosi & Dempsey, 1986) investigating glycogen utilization of the diaphragm in male rats supports minimal glycogen use during normoxic exercise (until exhaustion), yet glycogen utilization increased significantly in the diaphragm during hypoxic exercise. Decreased O_2_ transport, increased demand of the respiratory system, and increased circulating catecholamines during hypoxic exercise are significant changes that can impact glycogenolysis in both the respiratory and locomotor muscles (Fregosi & Dempsey, 1986). Males tend to have a higher reliance upon glycogen utilization globally during normoxic exercise (Tarnopolsky et al., 1990), and with decreasing F_I_O_2_, both males and females potentially increased overall glycogen metabolism, leading to greater metabolite accumulation and recovery rates. Future investigations of substrate utilization in males and females during exercise in hypoxia are needed.

### 4.4 Diaphragm force production during exercise

In both FiO_2_ conditions, the contribution of the diaphragm to total respiratory muscle force production (estimated by PTP_di_:PTP_eso_) decreased over time (Figure 3e). No sex‐based differences in PTP_di_:PTP_eso_ were observed, suggesting a similar contribution of the diaphragm to overall ventilation between males and females. However, PTP_di_:PTP_eso_ was significantly greater in normoxia than hypoxia at 80%TTE and 100%TTE, suggesting a greater contribution of the diaphragm in normoxia at the aforementioned time points. PTP_di_:PTP_eso_ slope was similar between males and females across conditions (Figure 3e), suggesting little effect of sex or FiO_2_ on the contribution of the diaphragm over time during exhaustive exercise. This finding contrasts that of Guenette et al. (2010), who observed a sex‐based difference in PTP_di_:PTP_eso_ slope during heavy exercise in endurance‐trained athletes. Reasons to explain the difference are not immediately obvious but may relate to our participants being "trained" whereas Guenette et al. (2010) studied highly‐trained cyclists with a high aerobic capacity. It is possible that specific habituation to cycle exercise may contribute to respiratory muscle recruitment.

### 4.5 Limitations

We (Guenette et al., 2010) have previously acknowledged the potential limitations of using CMS to stimulate the phrenic nerves. However, in the present study and in response to increasing magnetic stimulator intensity, we observed that P_di,tw_ plateaued at approximately 90% of maximal output for all groups. While this finding suggests maximal or near‐maximal stimulation of the phrenic nerves, we cannot exclude the possibility that stimulation was less than maximal. We did not control for menstrual cycle phase and tested females randomly throughout their cycle. Experimental days 2 and 3 were scheduled a maximum of 4 days apart in an attempt to decrease between‐day variation in hormone fluctuations, with the intention of having the testing days in the same phase of the cycle for each female. While the between‐subject variation in hormonal status may have influenced our findings, previous work from our group (Macnutt et al., 2012) demonstrated no effect of menstrual cycle phase on the ventilatory response to normoxic and hypoxic exercise. As such, the effects of cycle phase were unlikely to have had substantial effects on the ventilatory response to exercise in the present study.

## 5 CONCLUSION

We found that the magnitude of exercise‐induced diaphragm fatigue in males and females was not augmented by lowering F_I_O_2_. Experiencing the same degree of diaphragm fatigue in a shorter time period in acute hypoxic exercise versus normoxic exercise may relate to a decreased O_2_ transport to the diaphragm and/or increased levels of circulating metabolites. We also observed that females did not fully recover from diaphragm fatigue in hypoxia, whereas males did, which might relate to sex‐based differences in substrate utilization or diaphragm blood flow. Further study is required to determine if our observations extend to ambient hypoxia (e.g., high‐altitude) or disease states such as chronic obstructive pulmonary disease or heart failure.

## AUTHOR CONTRIBUTIONS

All authors contributed to interpretation of data and results, and revisions of intellectual content. All authors critically revised the manuscript for important intellectual content. All authors have approved the final version of the manuscript. All persons designated as authors qualify for authorship, and all those who qualify for authorship are listed.

## FUNDING INFORMATION

This work was funded by the Natural Sciences and Engineering Research Council of Canada (NSERC). The funders had no role in the study design, data collection and analysis, or preparation of the manuscript.

## CONFLICT OF INTEREST

No conflict of interest, financial or otherwise, are declared by the authors.
